# The Impact of Payment Reforms on the Quality and Utilisation of Healthcare for Patients With Multimorbidity: A Systematic Review

**DOI:** 10.5334/ijic.5937

**Published:** 2022-02-10

**Authors:** Toine E. P. Remers, Nina Nieuweweme, Simone A. van Dulmen, Marcel Olde Rikkert, Patrick P. T. Jeurissen

**Affiliations:** 1 Radboud University Medical Center, Radboud Institute for Health Sciences, Scientific Center for Quality of Healthcare (IQ healthcare), Nijmegen, NL; 2 Radboud University Medical Center, Radboud Institute for Health Sciences, Department of Geriatrics, Radboudumc Alzheimer Center, Nijmegen, NL

**Keywords:** multimorbidity, payment reform, quality of care, healthcare utilisation, healthcare financing, integrated care

## Abstract

Inadequate treatment of multimorbidity is recognised as a major determinant of the effectiveness of healthcare and also of its inappropriate expenditures. However, current payment systems target, primarily, the treatment of single diseases, thus hindering integrated delivery of care for patients with multimorbidity (PwM). This review aims to assess the effects of targeted reforms of payment systems which could help attain a higher quality of care and reduce unnecessary healthcare utilisation. In June 2020, a search of Medline and EMBASE revealed 13 relevant articles. The most common payment models were the use of bundled payments (n = 4) and diagnosis-related group payments (n = 4). Except for an increase in hospital admissions (n = 3), no outcome showed unambiguous significant effects across more than one study. The two studies which focused explicitly on PwM showed a significant decrease in 30-day hospital readmissions. This, however, was not maintained after 60 days in one study. No general conclusion could be drawn on the effects of targeted payment reforms for PwM. Our findings suggest that reforms should be combined with more multifaceted healthcare delivery to address the complex patterns of healthcare use effectively. Thorough evaluations of targeted payment reforms are needed urgently to contribute to the body of evidence required.

## Background

Multimorbidity, defined as the presence of multiple diseases or conditions, is a core topic in the field of healthcare management and is frequently the target of measures to increase the efficiency of healthcare systems [1,2,3,4]. Several systematic literature reviews have shown increasing prevalence of multimorbidity [1,5,6]. The complex pattern of healthcare use of such patients often results in poor coordination, leading to avoidable or insufficient care. Multimorbidity may ultimately result in ineffective and burdensome healthcare, thus giving rise to additional healthcare costs [7,8,9,10]. Nevertheless, current models for the delivery and payment are typically oriented towards the treatment of single diseases and are not customised to the healthcare needs of patients with multimorbidity (PwM) [1,2,11].

Although recent organisational reforms should facilitate the integrated delivery of care for PwM [12,13,14,15,16], Tinetti et al. argue that the current financial structure of healthcare systems hinders the success of such initiatives [17]. Hoedemakers et al. and Barnett et al. among others, support this view [1,18]. They state that such initiatives are mostly implemented in delivery systems that provide specific reimbursements for treating a certain condition [1,18]. Applying integrated care initiatives in payment systems that focus mainly on the treatment of a single disease might be ineffective because of undesired effects such as skimming off the easiest patients to treat and cost shifting. In such systems, introducing a payment reform designed to target PwM could contribute to a higher quality of care and, potentially, could reduce the healthcare utilisation for these groups of patients.

Studies testing this hypothesis have been conducted in various countries and settings, but large differences in populations, methods, and outcome measures exist [19,20,21,22,23]. Additionally, studies which systematically assess the results of such programmes focus solely on patients with one chronic condition. Such programmes are unlikely to address, sufficiently, the complex patterns of care concerning PwM that often accompany a primary condition [24,25]. Furthermore, these studies merely include outcome measures on either the quality or utilisation of healthcare.

Therefore, this systematic review seeks payment reforms which were designed to target PwM in order to assess their effects on both the quality and utilisation of healthcare with regard to PwM.

## Methods

### Search strategy

Medline and EMBASE, being the most widely used databases for peer-reviewed research articles, were chosen to search for relevant articles using a search string developed under the supervision of a medical information specialist. The search string consisted of terms related to three main topics: (i) payment reform, (ii) patients suffering from multimorbidity (see definition below), and (iii) outcomes regarding the quality and utilisation of healthcare. In particular, a filter excluded articles published before 01/01/2000. A detailed overview of the search strategy is presented in Appendix 1. Reference lists of the articles included were also screened to identify additional articles eligible for inclusion.

All types of payment systems or relevant outcome measures were included in the search. This applied both for payment reforms and the outcomes regarding the quality and utilisation of healthcare. This allowed for very broad terms to be included supplemented by well-known payment models and outcomes related to (i) and (iii), respectively. For (ii), multimorbidity emerged as a concept that has not yet been uniformly defined in medical and health services literature. Thus, we developed a search proxy in order to determine studies describing the outcomes of payment reforms for PwM. International comparisons have proved that older patients suffering from diabetes, chronic obstructive pulmonary disease, depression, chronic heart failure, chronic kidney disease, and dementia are most likely to display patterns of multimorbidity and were therefore included in our search proxy [24,25]. This led to the inclusion of both broad terms for multimorbidity and specific terms related to any of the six aforementioned conditions.

### Eligibility criteria

Initially only a few randomised controlled trials (RCTs) studying the effect of payment reforms on the selected outcomes of interest were revealed. Therefore, all peer-reviewed studies, including quasi-experimental studies or RCTs that reported results, directly or indirectly, of a payment reform were now included. However, studies that were not based on original data, such as Markov models, were excluded due to uncertainty originating from outcomes based on assumptions. To account for the existing heterogeneity in outcome measures, studies were included only if they encompassed any outcome for both quality and utilisation of healthcare. Additionally, studies were only included if their target population was specifically mentioned as being PwM or patients with one of the six chronic conditions included in our study.

Lastly, only payment reforms oriented towards PwM were included in this review. Studies were excluded if they did not introduce incentives beneficial for PwM through stimulating integrated care processes. The inclusion of six specific chronic conditions, however, would most likely result in payment reforms concentrating on one specific disease which would not benefit PwM. Therefore, a clear distinction between these two types of payment reforms had to be made. This distinction was based on two requirements: 1) in cases where payment reforms introduced incentives linked to certain outcomes, these incentives had to transcend specific conditions. For example, in cases of pay for performance (P4P) programmes for patients with diabetes, studies that rewarded providers independently for carrying out lipid testing and retina eye examinations were excluded. However, articles that rewarded providers for the planning of a full medication review and holding self-management sessions for patients were included. Additionally, 2) in the case of programmes with allocated funding, the budget had to cover all medical costs related to patients for a certain episode or period of care regardless of the conditions included in the programme. For example, in the case of reforms that introduced periodic payments for all inpatient services for patients with chronic heart failure (CHF), studies were only included if their allocated budget also included costs not related to CHF.

This led to us defining the targeted payment reforms that were included in our review as any change in the payment model that stimulates an integrated means of delivering care for PwM. We defined three separate payment models designed to stimulate integrated care. These were: payments linked to patients or populations rather than to individual care; payments that reward improvements in outcomes that transcend specific diseases; and payments that are linked to the provision of a predetermined set of care activities (payment bundles), of which these activities incorporate elements addressing more than one condition.

### Study selection and data extraction

Titles and abstracts were independently screened by two review authors (TR and NN). The remaining full articles were then screened again, independently, against the eligibility criteria (***Table 1***) by the same two review authors. For studies that do not explicitly focus on PwM (i.e. one of the six chronic conditions only), both authors independently performed an in-depth analysis of the contents of the payment reform in order to assess if these programmes met our definition of a targeted payment reform. For example, in the case of a bundled payment programme for patients with COPD, both authors assessed if the contents of the payment bundle would only improve COPD care or could also improve the care of a co-existing disease. Any discrepancies in both types of screening were resolved through consensus or consultation with a third review author (SvD).

**Table 1:** Inclusion and exclusion criteria.


INCLUSION CRITERIA	EXCLUSION CRITERIA

Study concerns patients with at least one of the selected chronic illnesses (COPD, diabetes, depression, CHF, chronic kidney disease, or dementia) OR explicitly focuses on patients with multimorbidity	No full text available (conference abstract, poster presentation)

The payment reform under study explicitly targets patients with multimorbidity and/or introduces a payment structure that can be beneficial for patients with multimorbidity by stimulating integrated care	The study is not about a payment reform (e.g. organisational reform only)

Peer-reviewed study, retrospective and prospective (e.g. quasi-experimental study; RCT)	The payment reform does not stimulate the integrated delivery of care to the patients in that it does not comply with our definition of a targeted payment reform

Outcomes concern both the quality and utilisation of healthcare	The outcomes of the study concern only the quality or utilisation of healthcare

Published since 01/01/2000	No original data

Written in English, Dutch	


COPD: Chronic obstructive pulmonary disease, CHF: Chronic heart failure.

The data extraction was subsequently performed by one review author (NN) and checked by, and discussed with, the second review author (TR). We used a standardised, Microsoft Excel data extraction form, developed and cross-validated beforehand (Appendix 2). To ensure, further, that the study results were concerned with PwM, data were only extracted for those patients specifically listed as PwM, or being part of one of six patient groups included in our study. The data on the use of healthcare was divided into disease-specific, and all-cause healthcare utilisation. This is because the effects that transcend specific diseases are particularly interesting for this PwM group.

### Data synthesis and analysis

The payment reforms we identified were clustered by one review author (NN) and were appraised and discussed with the second review author (TR). Payment reforms were clustered into five categories: Pay for performance (P4P), bundle, diagnosis-related group, capitation, and global budget. ***Box 1*** describes these categories further. A detailed description of the study’s outcome measures concerning both the quality and utilisation of healthcare was extracted for each study. Lastly, similarities in outcome measures across studies were used to form outcome domains for both the quality and utilisation of healthcare. For example, for mortality and healthcare costs.

The results of studies we included regarding all the outcome domains were synthesised into four possible outcomes: increase, decrease, mixed results, or none, that is no effect on the study’s specific outcomes. ‘Increase’ or ‘decrease’ signifies that the study found this significant (p ≤ 0.05) effect in all outcomes related to a specific outcome domain. ‘Mixed’ was used for studies with varying outcomes within one domain. ‘None’ was used for studies that found no statistically significant effect (p ≤ 0.05) for any of the outcomes related to a specific outcome domain or that studies did not report on any significance.

**Box 1 Definitions of targeted payment reforms.**

**Pay for performance (P4P):** a payment system in which reimbursement for healthcare providers are linked, entirely or in part, to the performance against a predetermined set of performance indicators.

**Bundled payment (bundle):** a payment system in which healthcare providers, within and across domains, receive a fixed payment for a predetermined set of services or treatments that is delivered to patients during an episode of care.

**Diagnosis-related group (DRG):** a payment system in which a healthcare provider (mostly a hospital) receives a standardised payment for all inpatient services during an episode of care, for example 30, 60 or 90 days, related to a certain diagnosis.

**Capitation:** a payment system where healthcare providers receive a fixed payment per beneficiary for all services, regardless of actual treatment being delivered.

**Global budget:** a payment system in which healthcare providers receive a fixed lump sum amount for a set period of time (e.g. year) and a specific patient population, regardless of actual treatment(s) being delivered.

### The risk of bias assessment

The risk of bias in the articles selected was independently assessed by two review authors (TR and NN) on a study level. The RoB-2 [26] and ROBINS-I [27] were used for randomised clinical trials and all remaining studies, respectively. Both tools are widely used instruments consisting of several domains concerned with potential sources of bias in studies such as randomisation and selection bias (see appendix 4). The domains were rated independently and together comprise an overall risk of bias on the study level. Any discrepancies were resolved through consensus or consultation with the third review author.

## Results

### Article characteristics

**Figure 1:**
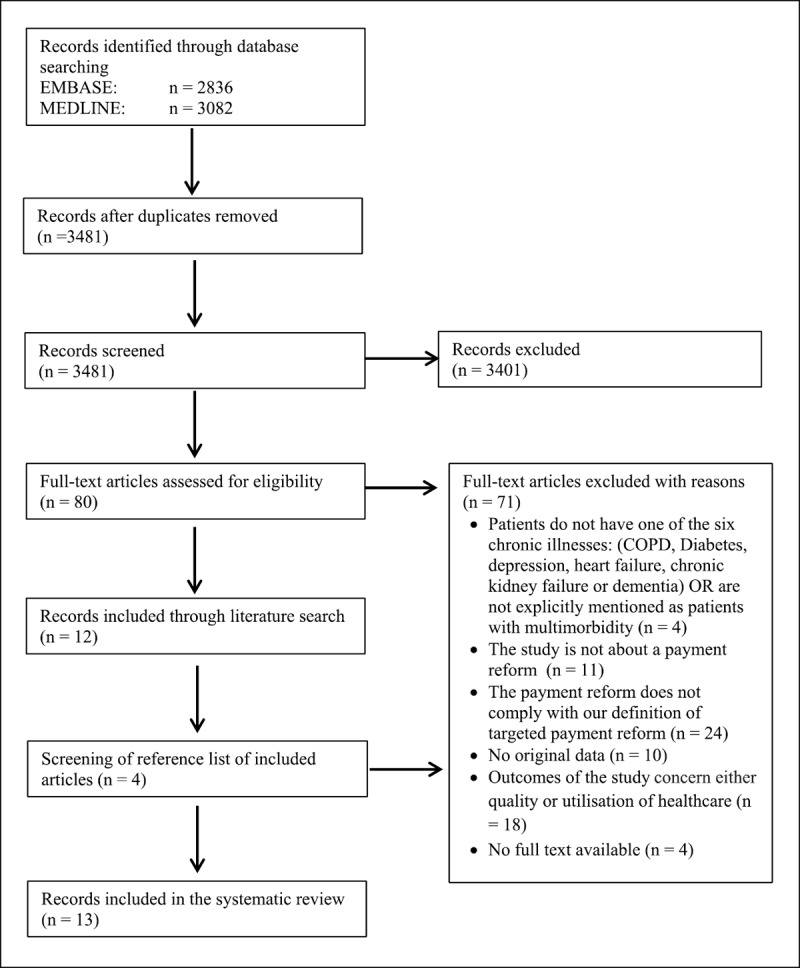
PRISMA flow diagram.

In total, 3,481 unique studies were identified through our search and were screened against the eligibility criteria. After the title or abstract screening, 80 articles were included for full-text screening. Finally, 13 articles were included. ***Figure 1*** demonstrates the PRISMA flow diagram. The main reason for excluding studies was that they did not comply with our definition of a targeted payment reform (n = 24). They were not, therefore, introducing a payment reform that would benefit PwM. Additionally, 18 studies were excluded because they solely reported outcomes related to either the quality or utilisation of healthcare.

***Table 2*** provides an overview of the study and programme characteristics sorted by payment reform. There were no studies that focused on patients with depression. Most studies focused on several groups of patients with chronic conditions, but only five studies addressed more than one of the six chronic conditions included in our search proxy for PwM. Moreover, only two of these studies explicitly mentioned focusing on PwM. Typically, the targeted payment reform under study represented a payment bundle (n = 4) or DRG (n = 4). Five out of these eight programmes were part of the same overarching Bundled Payments for Care Improvement or BPCI initiative. Other targeted payment reforms under study were capitation (n = 2), P4P (n = 2), and one using a global budget. All bundled payment and DRG programmes, as well as one capitation programme, were introduced in secondary care settings, whereas both P4P and the global budget programme were initiated in primary care. For one capitation programme, the payment reform was introduced across multiple settings. The new payment schemes were accompanied by an organisational reform in two studies.

**Table 2:** Study characteristics.


STUDY & COUNTRY [REFERENCE]	TARGET POPULATION	INTERVENTION	PROGRAMME CONTENTS	N (INTERVENTION, CONTROL)^*1^	SETTING	DATA COLLECTION PERIOD	STUDY TYPE (ANALYSES)	OUTCOMES

**Bhatt, S.P.** **United States [28]**	COPD	Bundle^*2^	Post-acute care bundle: antibiotics, educational materials, interval follow-up, and periodic phone calls	78, 109	Secondary (hospital) care	2012 vs. 2014	Cohort study with control group (independent sample statistical tests)	HR, EDV, Vis, HC, LoS

**Koehler, B.E.** **United States [29]**	PwM	Bundle	Elderly care bundle designed as an intensive patient-centred educational programme. Includes daily visits during hospital stay, standardised phone calls for follow-up appointments and education, and medication verification post discharge	22,20	Secondary (hospital) care	2007	Randomised control trial (independent sample statistical test)	HR, LoS

**Morton, K.** **United Kingdom [30]**	COPD	Bundle	COPD discharge bundle: technique (inhalers), action plan, pulmonary rehabilitation, smoking cessation, and specialist follow up.	4657, 4515	Secondary (hospital) care	2013–2017	Pre-post study with control group (regression models)	Mor, HR, EDV, LoS

**Parekh, T.M.** **United States [31]**	COPD	Bundle^*2^	Post-acute care bundle: expedited follow-up visits in a COPD focused clinic, home calls, medication assistance, and tobacco cessation counselling.	459, 239	Secondary (hospital) care	2012–2014	Cohort study with control group (independent sample statistical tests)	Mor, HR, EDV, HC, LoS

**Pawaskar, M.** **United States [32]**	Diabetes	Capitation	Managed care organisations receive a fixed amount of payment per enrolee per month	3763, 4818	Primary care, secondary (hospital) care	1999–2005	Cohort study with control group (regression models)	ADU, Hos, EDV, Vis

**Quinn, A.E.** **Canada [33]**	Diabetes, CKD	Capitation	A salary-like payment that covered clinical, research, and teaching time	15949, 15949	Secondary (hospital) care	2011–2015	Cohort study with matched control group (regression)	DRE, Hos, EDV, Vis, HC

**Joynt Maddox** **United States [21]** CHF, COPD		DRG^*2^	BPCI-model 2 bundle: Participating hospitals assume accountability for the costs of all care within 30, 60, or 90 days after hospitalisation for one or more of 48 conditions	226, 407 hospitals	Secondary (hospital) care	2013–2015	Pre-post study with matched control group (regression models)	Mor, HR, EDV, HC, LoS

**Kutz, A.** **Switzerland [34]**	COPD	DRG	All costs related to all acute inpatient hospital services	19046, 30764	Secondary (hospital) care	2009–2015	Pre-post study without control group (regression models)	Mor, HR, LoS

**Lichkus, J.** **United States [35]**	CHF	DRG^*2, 3^	One bundled payment per 90-day episode of care initiated by an anchor admission for CHF exacerbation.	283, 316	Secondary (hospital) care	2013–2017	Pre-post study without control group (t-tests)	HR, HC

**Maughhan, C.** **United States [36]**	Dementia	DRG^*2^	BPCI-model 2 bundle: Participating hospitals assume accountability for the costs of all care within 30, 60, or 90 days after hospitalisation for one or more of 48 conditions.	45007, 45007 episodes	Secondary (hospital) care	2011–2012 & 2013–2016	Pre-post study with matched control group (regression models)	Mor, HR, EDV

**Salzberg, C.A.** **United States [37]**	Diabetes	Global budget^*3^	Primary care practices receive a monthly, risk-adjusted total payment for the comprehensive care of all patients in the practice	64471, 133345	Primary care	2008–2013	Pre-post study with matched control group (regression models)	HR, Hos, EDV, Vis, HC

**Cross, D.A.** **United States [38]**	PwM	P4P	P4P-programme with incentives linked to 1) Medical Home Practice Transformation, 2) Provider-delivered Care Management, and 3) Practice Quality Assessment	17501, 195344	Primary care	2010–2013	Cohort study with control group (regression models)	DRE, ADU, HR, Hos, EDV, Vis, HC

**Hollander, M.J.** **Canada [39]**	Diabetes, CHF, COPD	P4P	P4P-programme with incentives linked to the provision of guidelines-based care to patients with chronic conditions	176542, 209064	Primary care	2010–2011	Cohort study with matched control group (paired samples t-test)	HR, Hos, HC


*^1^ Total number included in analyses relevant to this study – patients unless indicated otherwise. *^2^ Part of Bundled Payments for Care Improvement (BPCI) Initiative. *^3^ Payment reform is accompanied by an organisational reform. COPD: Chronic obstructive pulmonary disease, PwM: Patients with multimorbidity, CHF: Chronic health failure, CKD: Chronic kidney disease. **DRE:** Disease related examination(s)/treatment(s), **ADU:** Appropriate drug use, **Mor:** Mortality, **HR:** Hospital readmissions, **Hos:** Hospitalisations, **EDV:** Emergency Department Visits, **Vis:** Visits, **HC:** Healthcare costs, **LoS:** Length of Stay.

Four domains were formed among the quality of care outcomes. These were: disease-related examination/treatment, appropriate drug use, mortality, and hospital readmissions. Among these four domains, hospital readmission was most often included among the studies (n = 11). Furthermore, five outcome domains were formed for the utilisation of healthcare These were: hospitalisations, emergency department visits, visits, total healthcare costs, and length of stay. Healthcare costs were most often included (n = 8), but the way authors used this outcome differed across studies. Four studies introducing a payment bundle or DRG included payments for an episode of care of 30–90 days, whereas the remaining four studies included annual costs. A detailed overview of the study outcome measures is outlined in Appendix 3.

### Risk of bias assessment

The full risk of bias assessment for all studies is presented in Appendix 4. Notably, six out of the 13 studies were determined to have at least a serious risk of bias. All other seven studies were determined to have a moderate risk of bias or, in case of the randomised controlled trial of Koehler et al, showed ‘*some concerns*’ of bias.

### Effects of targeted payment reforms

The outcomes on the quality of care, categorised according to targeted payment reforms, are depicted in ***Table 3***. Overall, no significant unambiguous effect was found for any of the included outcome measures being assessed by more than one study. Furthermore, outcomes within any of the outcome domains did not seem to depend on the type of targeted payment reform being introduced, or the setting in which it was introduced (i.e. primary or secondary care). Two studies showed contradicting results for appropriate drug use. However, a significant positive effect of introducing a targeted payment reform was found for disease-related examinations or treatments and mortality (see ***Table 3***). For hospital readmissions, a significant decrease was found in one study whilst mixed results were found in another instance. When excluding the results from all six studies with at least a serious risk of bias, only the significant increases in disease-related examinations or treatments, appropriate drug use, and decrease in all-cause hospital readmissions found by Cross et al. [38] – plus the mixed results by Koehler et al. [29] – were retained. In the case of Cross et al, results affirmed after the introduction of their P4P programme were a 1.6% and 3.0% increase in disease-related examinations or treatments and appropriate drug use and 19.9%, and 27.5% decrease in 30- and 90-day hospital readmissions. Koehler et al. reported a significant 20% decrease in 30-day hospital readmissions, but failed to show significant differences in 60-day hospital readmissions.

**Table 3:** Effects of targeted payment reforms on the quality of care outcomes.


STUDY	PAYMENT MODEL	DISEASE-RELATED EXAMINATION(S)/TREATMENT(S)	APPROPRIATE DRUG USE	MORTALITY	HOSPITAL READMISSIONS		RISK OF BIAS

					*AC*	*DR*	

***Bhatt, S.P.***	*Bundle*	*n.a.*	*n.a.*	*n.a.*	None	None	Critical

***Koehler, B.E.***	*Bundle*	*n.a.*	*n.a.*	*n.a.*	Mixed	*n.a.*	Some concerns

***Morton, K.***	*Bundle*	*n.a.*	*n.a.*	None	None	None	Moderate

***Parekh, T.M.***	*Bundle*	*n.a.*	*n.a.*	Decrease	None	*n.a.*	Serious

***Pawaskar, M.***	*Capitation*	*n.a.*	Decrease	*n.a.*	*n.a.*	*n.a.*	Serious

***Quinn, A.E.***	*Capitation*	None	*n.a.*	*n.a.*	*n.a.*	*n.a.*	Moderate

***Joynt Maddox, K.E.***	*DRG*	*n.a.*	*n.a.*	None	None	*n.a.*	Serious

***Kutz, A.***	*DRG*	*n.a.*	*n.a.*	None	None	*n.a.*	Moderate

***Lichkus, J.***	DRG^*1^	*n.a.*	*n.a.*	*n.a.*	None	*n.a.*	Critical

***Maughhan, B.C.***	*DRG*	*n.a.*	*n.a.*	None	None	*n.a.*	Moderate

***Salzberg, C.A.***	Global budget ^*1^	*n.a.*	*n.a.*	*n.a.*	None	*n.a.*	Moderate

***Cross, D.A.***	*P4P*	Increase	Increase	*n.a.*	Decrease	*n.a.*	Moderate

***Hollander, M.J.***	*P4P*	*n.a.*	*n.a.*	*n.a.*	None	*n.a.*	Serious


***AC:*** All-cause, ***DR:*** Disease-related, ***n.a.*** Not applicable. *^1^ Payment reform is accompanied by an organisational reform. Increase’ or ‘decrease’ signifies that the study found a significant (p ≤ 0.05) effect in all outcomes related to a specific outcome domain. ‘Mixed’ was used for studies with varying outcomes within one domain. ‘None’ was used for studies that found no statistically significant effect (p ≤ 0.05) for any of the outcomes related to a specific outcome domain or that studies did not report on any significance.

Outcomes on healthcare utilisation sorted by targeted payment reform are reported in ***Table 4***. Overall, a significant increase in hospital admissions was found in three out of five studies. All other outcome measures mostly denote no effects or mixed results. When excluding the results of studies with at least a serious risk of bias, only Salzberg et al. [37] and Cross et al. [38] found significant increases in hospital admissions. Salzberg et al. specify a significant increase of 0.70 monthly admissions per 1,000 patients and Cross et al. indicate a 5.7% increase in admissions over three years compared to non-participants. The outcomes of these five studies do not seem to be dependent on the type of targeted payment reform being introduced, but all three studies that show an increase in hospital admissions were implemented in primary care settings.

**Table 4:** Effects of targeted payment reforms on outcomes related to the utilisation of healthcare.


STUDY	PAYMENT MODEL	HOSPITALISATIONS		ED VISITS		VISITS		HEALTHCARE COSTS		LENGTH OF STAY	RISK OF BIAS

		*AC*	*DR*	*AC*	*DR*	*AC*	*DR*	*AC*	*DR*		

**Bhatt, S.P.**	*Bundle*	*n.a.*	*n.a.*	*n.a.*	*n.a.*	*n.a.*	*n.a.*	None	*n.a.*	*n.a.*	Critical

**Koehler, B.E.**	*Bundle*	*n.a.*	*n.a.*	*n.a.*	*n.a.*	*n.a.*	*n.a.*	*n.a.*	*n.a.*	None	Some concerns

**Morton, K.**	*Bundle*	*n.a.*	*n.a.*	*n.a.*	None	*n.a.*	*n.a.*	*n.a.*	*n.a.*	None	Moderate

**Parekh, T.M.**	*Bundle*	*n.a.*	*n.a.*	Decrease	*n.a.*	*n.a.*	*n.a.*	Decrease	*n.a.*	Decrease	Serious

**Pawaskar, M.**	*Capitation*	Increase	*n.a.*	Increase	*n.a.*	Decrease	*n.a.*	*n.a.*	*n.a.*	*n.a.*	Serious

**Quinn, A.E.**	*Capitation*	None	*n.a.*	None	*n.a.*	None	*n.a.*	None	*n.a.*	*n.a.*	Moderate

**Joynt Maddox, K.E.**	*DRG*	*n.a.*	*n.a.*	None	*n.a.*	*n.a.*	*n.a.*	None	*n.a.*	None	Serious

**Kutz, A.**	*DRG*	*n.a.*	*n.a.*	*n.a.*	*n.a.*	*n.a.*	*n.a.*	*n.a.*	*n.a.*	None	Moderate

**Lichkus, J.**	DRG^*1^	*n.a.*	*n.a.*	*n.a.*	*n.a.*	*n.a.*	*n.a.*	None	*n.a.*	*n.a.*	Critical

**Maughhan, B.C.**	*DRG*	*n.a.*	*n.a.*	None	*n.a.*	*n.a.*	*n.a.*	*n.a.*	*n.a.*	*n.a.*	Moderate

**Salzberg, C.A.**	Global budget ^*1^	Increase	*n.a.*	None	*n.a.*	None	*n.a.*	None	*n.a.*	*n.a.*	Moderate

**Cross, D.A.**	*P4P*	Increase	*n.a.*	None	*n.a.*	None	*n.a.*	None	*n.a.*	*n.a.*	Moderate

**Hollander, M.J.**	*P4P*	None	*n.a.*	*n.a.*	*n.a.*	*n.a.*	*n.a.*	Increase	*n.a.*	None	Serious


***ED:*** Emergency Department, ***AC:*** All-cause, ***DR:*** Disease-related, ***n.a.*** Not applicable. * ^1^ Payment reform is accompanied by an organisational reform. Increase’ or ‘decrease’ signifies that the study found a significant (p ≤ 0.05) effect in all outcomes related to a specific outcome domain. ‘Mixed’ was used for studies with varying outcomes within one domain. ‘None’ was used for studies that found no statistically significant effect (p ≤ 0.05) for any of the outcomes related to a specific outcome domain or that studies did not report on any significance.

## Discussion

### Summary of findings

To our knowledge, this systematic review is the first to evaluate the impact of targeted payment reforms on the quality and utilisation of healthcare for PwM. Although the 13 studies we included were deemed to be targeted payment reforms, only the studies from Cross et al. [38] and Koehler et al. [29] mentioned that they were explicitly designed with a focus on PwM.

A significant increase in hospital admissions following the introduction of a targeted payment reform was found in three studies, of which two were determined to have a moderate risk of bias [37,38]. A significant decrease in 30-day hospital readmissions was found in both of the studies explicitly focusing on PwM [29,38]. However, the study by Koehler et al. did not show these effects continuing as they found no significant differences in 60-day hospital readmissions [29]. By contrast, the study by Cross et al. did demonstrate these effects continued, resulting in decreased 90-day hospital readmissions and beneficial effects across several other quality of care domains [38].

### The implications of the findings

No general conclusion could be drawn on the effect of targeted payment reforms for PwM due to the limited number of studies found, the heterogeneity in results, and because six out of the 13 studies included had at least a serious risk of bias. Despite this absence of unambiguous effects, a number of conclusions could be drawn from this systematic review.

First, the two studies that focused specifically on PwM [29,38] show beneficial effects, raising the possibility that targeted payment reforms might work in improving care for PwM. Both studies also support this notion by stating that these patients often have complex patterns of care, causing high rates of unplanned care which then require an integrated approach taking into account the different conditions that coexist [29,38]. Of the 11 remaining studies that were determined to be beneficial for PwM, despite not directly mentioning these patients, only four studies focused explicitly on more than one of the six chronic conditions shown to be strongly associated with multimorbidity. Others start with one primary disease, for example chronic obstructive pulmonary disease, and were determined to be beneficial for PwM, while not making the focus on this group of patients explicit. The absence of an improvement in both the quality and utilisation of healthcare for most of these programmes could indicate that such a focus might not have elicited a sufficient degree of integration in care for PwM. Nicholson et al. support this notion by stating that comorbidity and multimorbidity are often used interchangeably, while they mean two different things and require divergent approaches [40]. Comorbidity can be dealt with using disease specific programmes with additional components for coexisting complications. However, the best management of multimorbidity requires integrated care processes that are explicitly not centred on one primary chronic disease [40]. Previous research has shown disease-related incentives centred on one chronic disease can improve the quality of care of these programmes [41]. The beneficial results found in this review can be seen as the first step towards discovering mechanisms based on incentives and focused on the PwM. This is particularly important given the absence elsewhere of effective incentives needed to improve healthcare for PwM [1,42].

Furthermore, both studies that explicitly focus on PwM, highlight the importance of a multifaceted approach when attempting to improve healthcare for PwM. While current initiatives are hindered by the reimbursement structure in healthcare systems [17], both Cross et al. and Koehler et al. demonstrate that solely changing the financial structure does not seem to address the problem satisfactorily. In fact, they both state that wider approaches are necessary. Cross et al, for example, observed an increase in hospital admissions among PwM as follows: ‘*Significantly improving these outcomes, even among high-need patients who offer the greatest opportunity for gains, likely requires broader changes to the health system and to patient behaviour—both of which are complex and require a long time frame to address*’ [38]. Koehler et al. additionally states in relation to the diminishing effects on reducing readmission rates 30 days after discharge: ‘*An optimal intervention would capitalize on the hospital staff’s ability to improve short-term readmission/ED visit rates while linking patients to longer-term transitional care to extend these outcomes*’. Payment reforms should therefore be accompanied by successful organisational reforms that together are able to remove the existing barriers in healthcare systems to caring for PwM. Examples of such successful multifaceted approaches exist but most do not incorporate any change in financial structure and outcomes related to healthcare utilisation [13,17]. Yet, combined reforms are scarce in the scientific literature. In our opinion, the existing differences in procedures and regulations accompanying a change in either the organisation of healthcare or the payment system play a cardinal role in facilitating this scarcity of combined reforms. Whereas a payment reform is often initiated by a regulator or payer, such as a national and/or federal government or healthcare insurance company, organisational reforms can be put forward within the context of one’s organisation, since it does not necessarily require the commitment of regulators or payers. A combined reform therefore requires joint actions by healthcare regulators, payers, and providers to align procedures and regulations, and, in doing so, provide the necessary commitment on all levels.

Lastly, besides any possible implications related to the beneficial effects identified in this review, the limited number of payment reforms for PwM, and absence of their effects, could also simply express the inefficiency of payment reforms targeted at PwM. However, rather than these programmes simply being ineffective, it is more likely the problems arise from the high degree of complexity in translating multimorbidity into funding. Karimi et al. [43], for example, showed that a bundled payment programme focused on patients with chronic diseases resulted in additional costs for this group of patients as it failed to address the needs of PwM adequately. Default incentives or widely recognised good practices which target PwM specifically could help to reduce such inefficiencies and difficulties in the implementation, but are still mostly lacking [2].

### Strengths and limitations

To the best of our knowledge, this review is the first to focus on the effects of payment reforms on outcomes related to both the quality and utilisation of healthcare by PwM. This decision to include only articles that address both aspects has been made in an attempt to capture both the intended and unintended consequences of payment reforms, such as cost shifting. PwM are specifically susceptible to such unintended behaviour since their healthcare patterns are intricate. Additional efforts are required from providers to reach targets in comparison to patients with one chronic disease [44,45]. Whereas such unintended behaviour might cause some beneficial results in the utilisation of healthcare, other outcomes related to the quality of healthcare are probably affected negatively. In fact, two of the studies we included explicitly mention such behaviour and its negative consequences on the quality of healthcare of PwM [31,32].

Our decision to widen the definition of PwM with six chronic conditions resulted in the inclusion of payment reforms that focused on patients suffering from one or several of these six conditions. It is debatable if these payment reforms elicited beneficial effects for PwM. Additionally, this decision might have resulted in the exclusion of broad payment reforms that could have had beneficial effects. However, if we were to find any articles studying the effects of targeted payment reforms on PwM then the addition of these search terms was unavoidable given the absence of a uniform definition of multimorbidity in medical and health services literature. Moreover, a strict definition of what was considered to be a targeted payment reform was developed before inclusion and then all the studies we included were critically reviewed following this definition. Put all together, this strategy should have resulted in the inclusion of payment reforms targeting PwM through their ability to improve both the quality and utilisation of healthcare for these patients. This remains true even though they may not explicitly state this objective.

The heterogeneity in outcome measures did not enable us to draw firm conclusions about the clinical significance of outcomes related to targeted payment reforms. Instead, we now had to use statistical significance and adopted a cut-off value of p ≤ 0.05 for all effects to be determined as ‘increase’ or ‘decrease’. Such an approach may have resulted in the exclusion of very relevant, though not statistically significant data. Applying such a cut off, in this case, did, however, enable us to make comparisons and provided the first insight into the effect of targeted payment reforms for PwM in the absence of homogenous clinical outcomes across studies.

### Implications for practice and research

Several recommendations can be made based on the results of our study. First, we recommend that policymakers take the complex pattern of healthcare use by PwM into account when reforming the healthcare system in order to address their needs better. Policymakers should aim to implement multifaceted approaches, as previously discussed, in order to remove existing barriers in healthcare systems for PwM. Secondly, such approaches should consist of an appropriately targeted payment reform, leading to incentives targeted at PwM. However, it should also be combined with multifaceted approaches in healthcare delivery. Thirdly, regulators and payers, such as national/federal governments and healthcare insurers, should work together closely with healthcare providers in such endeavours in order to overcome possible challenges that healthcare organisations might face during these complex processes.

This study shows the need for more detailed evaluation of targeted payment reforms and their publication in scientific literature. Although we have found beneficial effects of targeted payment reforms, this body of evidence is still limited to two articles and provides insufficient grounds to state with full certainty that targeted payment reforms are effective for PwM. The absence of effects in all the remaining studies could also indicate that these payment reforms do not closely elicit any effects. It is likely that many more, potentially promising, targeted payment reforms have been introduced in various settings. Yet, if evaluations of such reforms are not published in scientific literature, then these cannot contribute to this urgently needed body of evidence. It is therefore highly recommended that initiators of such targeted payment reforms do make the additional effort to evaluate their work in some detail and then to publish their results.

## Additional File

The additional file for this article can be found as follows:

10.5334/ijic.5937.s1 Appendices. Appendix 1 to 4.
