# Supplementary material for: The Impact of Payment Reforms on the Quality and Utilisation of Healthcare for Patients With Multimorbidity: A Systematic Review
Source: Int J Integr Care. 2022 Feb 10;22(1):10. doi: 10.5334/ijic.5937 (PMC8833260; doi:10.5334/ijic.5937)
Supplement: Appendices. — Appendix 1 to 4. [file ijic-22-1-5937-s1.pdf]

Appendix 1: Search strategy

Medline

#	**Searches**
1	(Reimburs* OR payment OR pay-for-coordination OR pay for performance OR P4P OR budget OR capitation OR capitated).ab,ti,kf. OR (bundle* adj8 payment).ab,ti,kf. OR (bundle* adj8 care).ab,ti,kf.
2	exp Pulmonary Disease, Chronic Obstructive/ OR (COPD OR chronic obstructive pulmonary disease OR emphysema OR chronic bronchitis OR COAD OR Chronic Obstructive Airway Disease OR Chronic Obstructive Lung Disease OR Chronic Airflow Obstruction).ab,ti,kf.
3	exp Diabetes Mellitus/ OR (diabetes).ab,ti,kf.
4	exp Depressive Disorder/ OR (depression OR depressive disorder*).ab,ti,kf.
5	exp Heart Failure/ OR (chronic heart disease OR CHF OR heart failure OR cardiac failure).ab,ti,kf.
6	exp Renal Insufficiency, Chronic/ OR (chronic kidney disease OR Chronic renal insufficiency).ab,ti,kf.
7	exp Dementia/ OR (dementia OR Alzheimer*).ab,ti,kf.
8	exp Comorbidity/ or (multimorbidity or comorbidity).ab,ti,kf.
9	exp quality improvement/ OR (Quality OR Assessment of chronic illness care OR ACIC OR Patient Assessment of Chronic Illness Care OR PACIC OR QoL OR HRQoL).ab,ti,kf.
10	(Cost OR Costs OR Expenditure* OR hospitalization* OR hospitalisation OR Utili#ation OR spending OR rehospitali#ation*).ab,ti,kf.
11	#1 AND (#2 OR #3 OR #4 OR #5 OR #6 OR #7 OR #8) AND (#9 OR #10)
12	Filter: publication date ≥ 01/01/2000

EMBASE

#	**Searches**
1	(Reimburs* OR payment OR pay-for-coordination OR pay for performance OR P4P OR budget OR capitation OR capitated).ab,ti,kw. OR (bundle* adj8 payment).ab,ti,kw. OR (bundle* adj8 care).ab,ti,kw.
2	*chronic obstructive lung disease/ OR *chronic bronchitis/ OR *exp lung emphysema/ OR (COPD OR chronic obstructive pulmonary disease OR emphysema OR chronic bronchitis OR COAD OR Chronic Obstructive Airway Disease OR Chronic Obstructive Lung Disease OR Chronic Airflow Obstruction).ab,ti,kw.
3	*Diabetes Mellitus/ OR (diabetes).ab,ti,kw.
4	*exp depression/ OR (depression OR depressive disorder*).ab,ti,kw.
5	*****exp Heart Failure/ OR (chronic heart disease OR CHF OR heart failure OR cardiac failure).ab,ti,kw.

6	*chronic kidney failure/ OR (chronic kidney disease OR Chronic renal insufficiency).ab,ti,kw.
7	*exp dementia/ OR (dementia OR Alzheimer*).ab,ti,kw.
8	*exp multiple chronic conditions/ or *comorbidity/ or (multimorbidity or comorbidity).ab,ti,kw.
9	(Quality OR Assessment of chronic illness care OR ACIC OR Patient Assessment of Chronic Illness Care OR PACIC OR QoL OR HRQoL).ab,ti,kw.
10	(Cost OR Costs OR Expenditure* OR hospitalization* OR hospitalisation OR Utili#ation OR spending OR rehospitali#ation*).ab,ti,kw.
11	#1 AND (#2 OR #3 OR #4 OR #5 OR #6 OR #7 OR #8) AND (#9 OR #10)
12	Filter: publication date ≥ 01/01/2000 & conference abstract status

Appendix 2: Data extraction form (information extracted per study)

**First author**	
**Year**	
**Title**	
**Country**	
**Study type**	
**Disease(s)/patients (target group)**	
**1st, 2nd, 3rd**	
**Intervention**	
**Innovative payment model**	
**Comparison**	
**Data collection period**	
**Number of patients in intervention group**	
**Number of patients in comparison group**	
**Type of analysis**	
**Primary outcome measure**	
**Secondary outcome measure (s)**	
**Secondary outcome(s); conclusion(s)**	
**Remarks**	

Appendix 3: detailed overview of included outcome measures

Quality of care

Study, Year	Disease related examination(s)/treatment(s)*	Drug use*	Mortality	Hospital readmissions	
				All-cause	*Disease related*
Bhatt, S.P. 2016				30 and 90 day all cause readmission rate	30- and 90-day readmissions to acute exacerbations of COPD
Koehler. B.E. 2009				30 and 60 day all cause readmission rate	
Morton, K. 2019			Mortality at 90 days since discharge, in hospital mortality for COPD admissions	28-day overall readmission	28-day and 90-day COPD readmission
Parekh, T.M. 2018			In hospital mortality	30- or 90-day all-cause readmission	
Pawaskar, M. 2010		Medication adherence			
Quinn, A.E. 2019	Guideline-recommended care delivery				
Joynt Maddox, K.E. 2018			Mortality at 30 & 90 days	Readmission at 30 & 90 days	
Maughhan, B.C. 2019			Proportions of beneficiaries with 90 day all-cause mortality	Proportions of beneficiaries with 90 day unplanned hospital readmissions	
Kutz, A. 2019			All-cause in hospital mortality	30-day readmission rates	
Lichkus, J. 2019				Readmission rate	
Salzberg,				Readmissions	

C.A. 2017					
Cross, D.A. 2017	Overall composite score composed of 17 individual measures	A 6-measure medication management<br><br>sub composite		30 and 90 day all cause readmission rate	
Hollander, M.J. 2015				Readmission rates	

Healthcare utilisation

**Study, Year**	**Hospitalisations**		**ED visits**		**Visits**		**Healthcare costs**		**LOS**
	All-cause	*Disease related*	*All-cause*	*Disease related*	*All-cause*	*Disease related*	*All-cause*	*Disease related*	
Bhatt, S.P. 2016							30- and 90-day health care costs		
Koehler. B.E. 2009									Length of stay for index hospitalisation (days)
Morton, K. 2019				Number of ED admissions for COPD per month					Length of stay
Parekh, T.M. 2018			Rate of intensive care unit admission				Total costs of index admission		Hospital length of stay
Pawaskar, M. 2010	Hospitalisations		Emergency room visits		Outpatient visits				
Quinn, A.E. 2019	Rate of hospital admissions or ED visits		Rate of hospital admissions or ED		Follow-up outpatient visits		Mean total costs		

			visits						
Joynt Maddox, K.E. 2018			Emergency department use at 30 & 90 days				Total 90 day Medicare payments		Mean length of stay
Maughhan, B.C. 2019			Proportions of beneficiaries with 90 day ED visits						
Kutz, A. 2019									Length of stay
Lichkus, J. 2019							Total episode cost per patient		
Salzberg, C.A. 2017	Number of inpatient admissions		The number of ED visits		Outpatient office visits		Medical expenditures per member per month		
Cross, D.A. 2017	Number of inpatient admissions		Visits		Primary care Physician & specialist visits		Total allowed medical surgical costs per member per year		
Hollander, M.J. 2015	Net number of admissions						Total costs		Average length of stay per regular admission, hospital stay and hospital stay episode
ED: **emergency department**									

Appendix 4: Risk of bias assessment

ROBINS-I

Study, Year	Baseline confounding	Selection bias	Classification of intervention	Deviation from intended intervention	Missing data	Measurement of outcomes	Selection of reported results	Overall risk of bias
Bhatt, S.P. 2016	Critical	Low	Low	Low	No information	Low	Moderate	**Critical**
Morton, K. 2019	Moderate	Low	Low	Low	Moderate	Low	Low	**Moderate**
Parekh, T.M. 2018	Serious	Low	Low	Serious	Low	Low	Moderate	**Serious**
Pawaskar, M. 2010	Moderate	Serious	Low	Low	Low	Low	Moderate	**Serious**
Quinn, A.E. 2019	Moderate	Low	Low	Low	Low	Low	Moderate	**Moderate**
Joynt Maddox, K.E. 2018	Moderate	Serious	Low	Low	Low	Low	Moderate	**Serious**
Maughhan, B.C. 2019	Moderate	Low	Low	Low	Low	Low	Moderate	**Moderate**
Kutz, A. 2019	Moderate	Low	Low	Low	Low	Low	Moderate	**Moderate**
Lichkus, J. 2019	Critical	Low	Low	Low	Low	Low	Moderate	**Critical**
Salzberg, C.A. 2017	Moderate	Low	Low	Low	Low	Low	Moderate	**Moderate**
Cross, D.A. 2017	Moderate	Low	Low	Low	Low	Low	Moderate	**Moderate**
Hollander, M.J. 2015	Serious	Serious	Low	Low	No information	Low	Moderate	**Moderate**

ROB-2

Study, Year	Randomisation process	Deviations from intended interventions	Missing outcomes	Measurement of the outcome	Selection of reported results	Overall risk of bias
Koehler B	Low	Low	Low	Low	Some concerns	**Some concerns**
